# Severe maternal outcomes in eastern Ethiopia: Application of the adapted maternal near miss tool

**DOI:** 10.1371/journal.pone.0207350

**Published:** 2018-11-14

**Authors:** Abera Kenay Tura, Joost Zwart, Jos van Roosmalen, Jelle Stekelenburg, Thomas van den Akker, Sicco Scherjon

**Affiliations:** 1 School of Nursing and Midwifery, College of Health and Medical Sciences, Haramaya University, Harar, Ethiopia; 2 Department of Obstetrics and Gynecology, University Medical Centre Groningen, University of Groningen, Groningen, the Netherlands; 3 Department of Obstetrics and Gynecology, Deventer Ziekenhuis, Deventer, the Netherlands; 4 Department of Obstetrics, Leiden University Medical Centre, Leiden, the Netherlands; 5 Athena Institute, VU University Amsterdam, Amsterdam, the Netherlands; 6 Department of Health Sciences, Global Health, University Medical Centre Groningen, University of Groningen, Groningen, the Netherlands; 7 Department of Obstetrics and Gynecology, Leeuwarden Medical Centre, Leeuwarden, the Netherlands; University of Michigan Medical School, UNITED STATES

## Abstract

**Background:**

With the reduction of maternal mortality, maternal near miss (MNM) has been used as a complementary indicator of maternal health. The objective of this study was to assess the frequency of MNM in eastern Ethiopia using an adapted sub-Saharan Africa MNM tool and compare its applicability with the original WHO MNM tool.

**Methods:**

We applied the sub-Saharan Africa and WHO MNM criteria to 1054 women admitted with potentially life-threatening conditions (including 28 deaths) in Hiwot Fana Specialized University Hospital and Jugel Hospital between January 2016 and April 2017. Discharge records were examined to identify deaths or women who developed MNM according to the sub-Saharan or WHO criteria. We calculated and compared MNM and severe maternal outcome ratios. Mortality index (ratio of maternal deaths to SMO) was calculated as indicator of quality of care.

**Results:**

The sub-Saharan Africa criteria identified 594 cases of MNM and all the 28 deaths while the WHO criteria identified 128 cases of MNM and 26 deaths. There were 7404 livebirths during the same period. This gives MNM ratios of 80 versus 17 per 1000 live births for the adapted and original WHO criteria. Mortality index was 4.5% and 16.9% in the adapted and WHO criteria respectively. The major difference between the two criteria can be attributed to eclampsia, sepsis and differences in the threshold for transfusion of blood.

**Conclusion:**

The sub-Saharan Africa criteria identified all the MNM cases identified by the WHO criteria and all the maternal deaths. Applying the WHO criteria alone will cause under reporting of MNM cases (including maternal deaths) in this low-resource setting. The mortality index of 4.5% among women who fulfilled the adapted MNM criteria justifies labeling these women as having ‘life-threatening conditions’.

## Introduction

With the reduction of maternal mortality, the study of women who survived life-threatening complications during pregnancy, childbirth and postpartum period has gained attention since the 1990s [1–3]. Severe acute maternal morbidity or maternal near miss (MNM) [1–6]—both referring to a woman surviving a clinical spectrum of severity—were used to refer to such survivors of life-threatening complications. To harmonize definition and identification of MNM, the World Health Organization (WHO) published the standard MNM tool in 2009 [7], followed by a guideline on how to apply the WHO MNM approach in 2011 [8]. According to WHO definition, MNM refers to a woman who nearly died but survived a life-threatening complication that occurred during pregnancy, childbirth or within 42 days of termination of pregnancy. Twenty-five criteria divided into three groups—clinical, laboratory based, and management based—were set as indicators for the presence of MNM [7].

The WHO MNM tool has been used in several MNM studies, including in low-income settings where the tool was found to lead to significant underreporting of serious illness [9–11]. In settings where the tool was applied without adaptation, the frequency of MNM was very low and almost equal to maternal deaths—minimizing clinical relevance of the tool [12,13]. For example, although for every maternal death, an estimated 20 maternal injury, infection, disease, or disability (including MNM) are expected [14], very low proportions are reported in several low-income settings: 1.3 in Zanzibar, 2.5 in Ghana, 1.5 in Nigeria, 6.1 in Tanzania, and 6.2 in South Africa [12,13,15–17]. But studies using adapted criteria or disease based criteria reported higher maternal near miss to mortality ratios [18,19].

The need for practical criteria for use in low-income settings was previously reported [10] and individual adaptations were suggested [9]. In order to improve the applicability of the WHO MNM tool for use in low-income settings, we developed a sub-Saharan Africa MNM tool as described previously [20]. In brief, forty-seven international experts rated the applicability of the WHO MNM criteria and suggested additional parameters over three rounds. Twenty-seven criteria (19 out of 25 original WHO criteria; and eight newly suggested ones) were agreed for use in low-income sub-Saharan Africa settings. This study presents findings from the application of the sub-Saharan Africa MNM tool in Ethiopia and discusses the differences in the applicability of this tool compared to the original WHO MNM tool.

## Materials and methods

### Study setting

This study was conducted from January 2016 to April 2017 in Hiwot Fana Specialized University Hospital (HFSUH) and Jugel Regional Hospital in Harar town. HFSUH is a tertiary referral hospital affiliated with the College of Health and Medical Sciences of Haramaya University, Ethiopia. It is the major referral hospital in the eastern part of the country serving a catchment area with a population close to 3 million. HFSUH has two major operation rooms—one for general cases and one specifically for obstetrics—and a central intensive care unit with standby generator for use during power breaks. The maternity unit, consisting of 41 beds, serves both referred and self-referred women. During the study period, the unit was run by seven consultants, eight residents, and more than 20 nurse midwives. One anesthesiologist was available in the hospital, based on a monthly rotation from the capital. Jugel Hospital is a regional general hospital found in the same town, run by the Harari Regional Health Bureau. The maternity unit was run by integrated emergency surgical officers (associate clinicians) [21] under the supervision of consultants from HFSUH. Since HFSUH is relatively well equipped (including the only neonatal intensive care unit and pediatric ward in the region), the majority of complications are referred to this hospital.

### Study design and participants

In this prospective cohort study, we included all women with MNM according to the sub-Saharan Africa or original WHO MNM criteria. Identification of MNM was a two-step process—we first identified all women with potentially life-threatening conditions (PLTC) as defined by WHO (severe postpartum hemorrhage, severe pre-eclampsia, eclampsia, uterine rupture, severe complications of abortion, and sepsis/severe systemic infections); received critical interventions (use of blood products, laparotomy other than cesarean section); or were admitted to the intensive care unit [8]. At discharge, we then selected those who developed life-threatening complications, consisting of MNM and maternal deaths, according to the sub-Saharan Africa or original WHO MNM criteria [8,20]. Maternal near miss refers to a woman who nearly died but survived a life-threatening complication that occurred during pregnancy, childbirth or within 42 days of termination of pregnancy [7]. Severe maternal outcome includes women with life-threatening complications who survived the complications (near miss) or died. Eligible women were identified by trained research assistant nurse-midwives working in both hospitals through daily visits of obstetric ward, intensive care unit, emergency room, and gynaecology ward. Identified cases were evaluated and confirmed by the first author (AKT). Sample size was estimated based on the annual deliveries and maternal mortality ratio according to the recommendation by the WHO [22]. Considering the existing maternal mortality ratio (412) and the annual number of deliveries in both hospitals, we expected 7000 live births and 30 maternal deaths in 16 months.

### Measurement and quality assessment

For all women with PLTC, or who received critical interventions, or admitted to the intensive care unit, basic identifying information (medical registration number, the underlying complication, and admission unit) were recorded daily and followed until discharge. Upon discharge, a thorough review of her medical record was conducted to collect detailed data on socio-demographic characteristics, history of morbidities, obstetric conditions, underlying complication, MNM event, treatments received, and maternal and perinatal outcomes. Information about referral status was also collected. Referred cases refers to women coming from health centers and district hospitals with existing complications. This enabled us to distinguish occurrence of MNM before or after admission—a good indicator of in hospital quality of care and referral system.

The dependent variable was presence of maternal near miss or maternal death. Maternal death was defined as a death of woman while pregnant or within 42 days of termination of pregnancy. Maternal near miss was identified by the presence of any of the life-threatening complications listed in Table 1. Independent variables included socio-demographic characteristics (age, referral status, residence), obstetric conditions (parity, place of delivery, gravidity, antenatal care, mode of delivery), underlying medical complications, and infection. Data about the total number of deliveries was obtained from monthly hospital reports. In case of doubt and when additional information was required, attending clinicians were contacted for clarification. The overall data collection and quality of data was supervised by the first author (AKT) and two experienced researchers from the College of Health and Medical Sciences, Haramaya University. All completed questionnaires were checked for completeness and consistency before entry to the computer. Codes were used to identify each woman included in the study and no personal identifiers were included in the analysis or reporting. Access to collected data was restricted only to the research team and the questionnaire was kept in locked cabinet.

**Table 1:** The adapted sub-Saharan Africa MNM tool.

WHO maternal near miss criteria	sub-Saharan Africa maternal near miss criteria
**Clinical criteria**	
Acute cyanosis	Acute cyanosis ^a^
Gasping	Gasping ^b^
Respiratory rate >40 or <6/min	Respiratory rate >40 or <6/min
Shock	Shock ^c^
Oliguria nonresponsive to fluids or diuretics	Oliguria nonresponsive to fluids or diuretics ^d^
Failure to form clots	Failure to form clots ^e^
Loss of consciousness lasting ≥12 h	Loss of consciousness lasting ≥12 h ^f^
Cardiac arrest	Cardiac arrest
Stroke	Stroke ^g^
Uncontrollable fit/total paralysis	Uncontrollable fit/total paralysis ^h^
Jaundice in the presence of pre-eclampsia	Jaundice in the presence of pre-eclampsia ^i^
	Eclampsia ^j^
	Uterine rupture ^k^
	Sepsis or severe systemic infection ^l^
	Pulmonary edema ^m^
	Severe abortion complications ^n^
	Severe malaria ^o^
	Severe pre-eclampsia with ICU admission
**Laboratory-based criteria**	
Oxygen saturation <90% for >60 minutes	Oxygen saturation <90% for >60 minutes
PaO2/FiO2 <200 mmHg	
Creatinine ≥300μmol/l or ≥3.5 mg/dl	Creatinine ≥300μmol/l or ≥3.5 mg/dl
Bilirubin >100 μmol/l or >6.0 mg/dl	
pH <7.1	
Lactate >5 mEq/ml	
Acute thrombocytopenia (<50,000 platelets/ml)	Acute thrombocytopenia (<50,000 platelets/ml)
Loss of consciousness and ketoacids in urine	Loss of consciousness and ketoacids in urine
**Management based criteria**	
Use of continuous vasoactive drugs	
Hysterectomy following infection or haemorrhage	Hysterectomy following infection or haemorrhage
Transfusion of ≥5 units of blood	Transfusion of ≥2 units of red blood cells
Intubation and ventilation for ≥60 min not related to anaesthesia	Intubation and ventilation for ≥60 min not related to anaesthesia
Dialysis for acute renal failure	
Cardio-pulmonary resuscitation	Cardio-pulmonary resuscitation
	Laparotomy other than caesarean section

^a^ Acute cyanosis is blue or purple colouration of the skin or mucous membranes due to low oxygen saturation

^b^ Gasping is a terminal respiratory pattern, and the breath is convulsively and audibly caught.

^c^ Shock is persistent severe hypotension, defined as a systolic BP <90 mmHg for ≥60 min with a pulse rate at least 120 despite aggressive fluid replacement (>2l)

^d^ Oliguria is urinary output < 30 ml/h for 4 h or < 400 ml/24 h

^e^ Failure to form clots can be assessed by the bedside clotting test or absence of clotting from the IV site after 7–10 minutes

^f^ Loss of consciousness lasting >12h is a profound alteration of mental state that involves complete or near-complete lack of responsiveness to external stimuli. It is defined as a Glasgow Coma Scale <10 (moderate or severe coma)

^g^ Stroke is a neurological deficit of cerebrovascular cause that persists beyond 24 h or is interrupted by death within 24 h

^h^ Uncontrolled fits/total paralysis is refractory, persistent convulsions or status epilepticus

^i^ Pre-eclampsia is defined as the presence of hypertension associated with proteinuria. Hypertension is defined as a BP of at least 140/90 mmHg on at least two occasions and at least 4–6 h apart after the 20th week of gestation in women known to be normotensive beforehand. Proteinuria is defined as excretion of 300 mg or more of protein every 24 h. If 24-h urine samples are not available, proteinuria is defined as a protein concentration of 300 mg/l or more (≥1 on dipstick) in at least two random urine samples taken at least 4–6 h apart.

^j^ Eclampsia is diastolic BP ≥90 mmHg or proteinuria +3 and convulsion or coma

^k^ Uterine rupture is complete rupture of uterus during labour and/or confirmed later by laparotomy

^l^ Sepsis or severe systemic infection is defined as a clinical sign of infection and 3 of the following: temp >38 ^0^C or <36°C, respiration rate >20/min, pulse rate >90/min, WBC >12,000

^m^ Pulmonary edema is accumulation of fluids in the air spaces and parenchyma of the lungs

^n^ Severe abortion complications is defined as septic in incomplete abortion, complicated Gestational Trophoblastic Disease with anaemia

^o^ Severe malaria is defined as major signs of organ dysfunction and/or high-level parasitemia or cerebral malaria

### Data processing and analysis

Data were entered using EpiData v3.1 (www.epidata.dk) and IBM SPSS Statistics for Windows, version 23 (IBM Corp., Armonk, N.Y., USA) was used for analysis. Descriptive statistics of study participants and indicators of MNM were analyzed. Severe maternal outcome ratio, MNM ratio, mortality index and MNM to mortality ratio were calculated. Severe maternal outcome ratio is the total number of women with life-threatening complications (MNM and maternal deaths) per 1000 live births. Similarly, MNM ratio refers to the total number of MNM per 1000 live births. Mortality index is the ratio of maternal deaths to the total number of women with life-threatening complications [5]. A lower mortality index level indicates good quality of care. The study was approved by the Institutional Health Research Review Committee of the College of Health and Medical Sciences, Haramaya University, Ethiopia (Ref N: C/A/R/D/01/1681/16). Since data were collected from medical charts after discharge of the women and no patient interview was planned, the need for informed consent was waived. Permission was obtained from the respective officials in the regional health bureau and participating hospitals.

## Results

**Fig 1:**
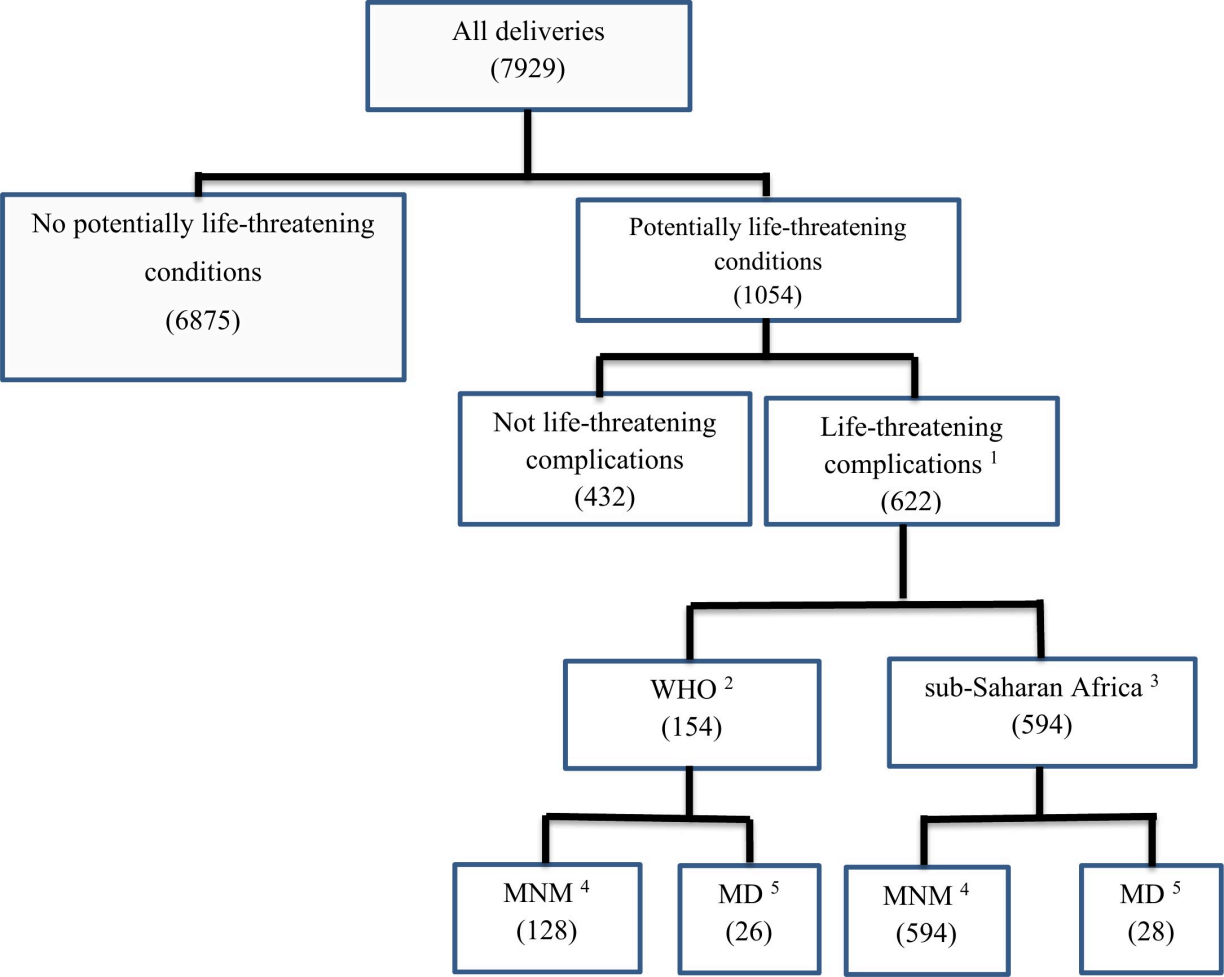
Study flow chart of severe maternal outcomes in eastern Ethiopia. ^1^According to the sub-Saharan or WHO criteria ^2^World Health Organization MNM criteria ^3^ sub-Saharan Africa MNM criteria ^4^Maternal Near Miss ^5^Maternal Deaths.

Of 1054 women admitted with potentially life-threatening conditions during the study period, 622 were classified as life-threatening complications by the sub-Saharan Africa criteria: 28 maternal deaths and 594 MNM. When the original WHO criteria was applied, 154 were classified as life-threatening complications: 26 maternal deaths and 128 MNM (Fig 1). During the same period, a total of 7929 deliveries and 7404 livebirths were registered in both hospitals, resulting in a maternal near miss ratio of 80 and 17 per 1000 live births according to the sub-Saharan Africa and WHO criteria respectively. The MNM ratio was 106 and 46 per 1000 livebirths in HFSUH and Jugel Hospital respectively. According to the WHO criteria, the MNM ratio was 29.4 and 5.9 per 1000 live births in HFSUH and Jugel Hospital respectively. All the 28 maternal deaths occurred in HFSUH.

### Characteristics of participants

Majority of the study participants were 20–35 years old, received no antenatal care, and referred from other facilities. No statistically significant difference was observed between MNM and deaths except referral status, which was higher among cases of maternal deaths than the maternal near miss (Table 2).

**Table 2:** Sociodemographic and obstetric characteristics of MNM and deaths in eastern Ethiopia.

Variables	MNM (n = 594)	MD (n = 28)	p-value
**Age mean (SD)**	**25.4(±6.1)**	**25.8(±6.1)**	
<20	76(12.9)	2(7.1)	0.606
20–35	485(82.2)	24(85.7)	
>35	29(4.9)	2(7.1)	
**Received antenatal care**			
Yes	172(29.2)	7(25.0)	0.636
No	418(70.8)	21(75)	
**Gestational age (weeks)**			
<28	29(5.6)	2(7.1)	0.537
28–36	167(31.9)	11(39.3)	
≥37	327(62.5)	14(50.0)	
**Parity**			
0	153(26.0)	6(21.4)	0.822
1–4	283(48.0)	15(53.6)	
>4	153(26.0)	7(25.0)	
**Mode of delivery**			
Vaginal	324(54.9)	16(57.1)	0.804
Cesarean section	166(28.2)	8(28.6)	
Laparotomy	46(7.8)	1(3.6)	
Abortion	32(5.4)	1(3.6)	
No delivery	22(3.7)	2(7.1)	
**Referred from other facility**			
Yes	361(61.1)	25(89.3)	0.003
No	230(38.9)	3(10.7)	
**Fetal outcome at birth**			
Alive	356 (76.9)	15(62.5)	0.103
Stillbirth	107(23.1)	9(37.5)	

MNM, maternal near miss; MD, maternal death; SD, standard deviation

The major difference in the number of MNM between the sub-Saharan Africa and the WHO MNM criteria can be attributed to eclampsia, sepsis, and differences in the threshold for the transfusion of blood (Table 3). The threshold in the sub-Saharan Africa is two units compared to five in the WHO criteria. Of 118 women who received only two units of blood, 87 have no other WHO inclusion criteria. Only nine women received five or more units of blood (S1 Fig). For two of the 28 maternal deaths which fulfilled the sub-Saharan African criteria (pulmonary edema and two units of blood), reported data were insufficient to fulfill the WHO criteria.

**Table 3:** Distribution of MNM according to the sub-Saharan and WHO criteria.

Parameter	SSA(n)	WHO(n)	Cases not fulfilling the WHO criteria (n)
**Maternal near miss**	**594**	**128**	**466**
**Maternal deaths**	**28**	**26**	**2**
**Clinical criteria**			
Acute cyanosis	3	3	0
Gasping	7	7	0
Respiratory rate >40 or <6/min	26	26	0
Shock	51	51	0
Oliguria nonresponsive to fluids or diuretics	2	2	0
Failure to form clots	13	13	0
Loss of consciousness lasting ≥12 hours	10	10	0
Cardiac arrest	3	3	0
Stroke	3	3	0
Uncontrollable fit/status epilepticus	6	6	0
Jaundice in the presence of pre-eclampsia	4	4	0
Eclampsia	227	26	201
Uterine rupture	53	39	14
Sepsis	129	19	110
Pulmonary edema	13	7	6
Severe complications of abortion	16	3	13
** Any clinical criteria**	**446**	**91**	**335**
**Laboratory-based criteria**			
Oxygen saturation <90% for >60 minutes	17	17	0
Creatinine ≥300μmol/l or ≥3.5 mg/dl	1	1	0
Acute thrombocytopenia (<50,000 platelets/ml)	14	14	0
** Any laboratory based criteria**	**27**	**27**	**0**
**Management-based criteria**			
Hysterectomy following infection or hemorrhage	55	55	0
Use of blood products ^a^	177	59	118
Intubation and ventilation for ≥60 min not related to anesthesia	13	13	0
Cardio-pulmonary resuscitation	10	10	0
Laparotomy other than for cesarean section	77	44	33
Severe pre-eclampsia with ICU admission	17	8	9
** Any management based criteria**	**266**	**77**	**189**
**Total severe maternal outcome** ^**b**^	**622**	**154**	**468**

SSA, sub-Saharan Africa; WHO, World Health Organization

^a^ Two or more units of blood

^b^ Total exceeds total number of cases since some women have more than one inclusion criteria

### Maternal near miss indicators

The MNM ratio was 80 per 1000 live births according to the sub-Saharan Africa criteria. For every maternal death, there were 21 MNM cases resulting in a mortality index of 4.5%. For the original WHO criteria, MNM ratio was lower (17 per 1000), mortality index was much higher (16.9%) and MNM to mortality ratio was lower (4.9:1) compared to the adapted criteria (21:1). A high proportion (85.2% in the adapted and 82.5% in the original criteria) of MNM was already present on arrival or occurred within 12 hours of admission, majority (87% in the adapted and 68% in the WHO criteria) of whom were referred from other facilities. Mortality index was almost double among referred cases compared to in-hospital MNM cases in both classifications (Table 4).

**Table 4:** Severe maternal outcomes and near-miss indicators in eastern Ethiopia, 2017.

Outcomes	Near-miss indicators	
	SSA	WHO
1. All live births in the population under surveillance	7404	7404
2. Severe maternal outcomes (SMO) cases (number)	622	154
Maternal deaths (n)	28	26
Maternal near-miss cases (n)	594	128
**3. Overall near-miss indicators**		
Severe maternal outcome ratio (per 1000 live births)	84	20.8
Maternal near-miss ratio (per 1000 live births)	80.2	17.3
Maternal near-miss mortality ratio (MNM:MD)	21.2	4.9
Mortality index (%)	4.5	16.9
**4. Hospital access indicators**		
SMO cases presenting the organ dysfunction or maternal death within 12 hours of hospital stay (SM012) (number)	530	127
Proportion of SM012 cases among all SMO cases	85.2	82.5
Proportion of SM012 cases coming from other health facilities	86.5	67.7
SM012 mortality index (%)	4.9	18.9
**5. Intrahospital care**		
Intrahospital SMO cases (number)	92	27
Intrahospital SMO rate (per 1000 live births)	12.4	0.4
Intrahospital mortality index (%)	2.2	7.4

SMO, severe maternal outcome; MNM, maternal near miss; MD, maternal death; SSA = sub-Saharan Africa; WHO, World Health Organization

Hypertensive disorders and obstetric hemorrhage were the major underlying complications in MNM and deaths. In the adapted tool, hypertensive disorders were the leading underlying complication while obstetric hemorrhage is common in the WHO criteria. Anemia was the leading contributory factor in both criteria. Details of underlying complications and associated factors are presented in Table 5.

**Table 5:** Underlying causes of life-threatening conditions and severe maternal outcomes in eastern Ethiopia.

Variables	Sub-Saharan Africa tool			WHO tool		
	MNM	MD	MI	MNM	MD	MI
	n (%)	n (%)	%	n (%)	n (%)	%
**Overall**	**594**	**28**	**4.5**	**128**	**26**	**16.9**
**Underlying complications**						
**Hypertensive disorders**	**271(45.6)**	**14(50)**	**4.9**	**36(28.1)**	**13(50)**	**26.5**
Severe pre-eclampsia	52(8.8)	6(21.4)	9.5	17(13.3)	5(19.2)	22.7
Eclampsia	219(36.9)	9(32.1)	3.6	18(14.1)	8(30.8)	30.8
**Obstetric hemorrhage**	**214(36.0)**	**14 (50)**	**6.3**	**79(61.7)**	**14(53.8)**	**15.1**
Abortion related	25(4.2)	1(3.6)	3.9	6(4.7)	1(3.8)	14.3
Ectopic pregnancy	21(3.5)	0(0)	0	3(2.3)	0(0)	0
Abruptio placenta	22(3.7)	4(14.3)	12	3(2.3)	3(11.5)	50
Placenta previa	24(4.0)	1(3.6)	4	11(8.6)	1(3.8)	8.3
Uterine rupture	46(7.7)	2(7.1)	4.2	34(26.6)	2(7.7)	5.6
Severe postpartum hemorrhage	48(8.1)	6(21.4)	12.5	16(12.5)	8(30.8)	33.3
Others	18(3.0)	0(0)	0	1(0.8)	0(0)	0
**Sepsis/severe systemic infection**	**126(21.2)**	**3(10.7)**	**2.4**	**20(15.6)**	**5(19.2)**	**20**
**Contributory factors**						
Anemia	195(32.8)	14(50)	6.7	52(40.6)	13(50)	20
Previous cesarean section	15(2.5)	1(3.6)	6.3	5(3.9)	1(3.8)	16.7
**Critical interventions**						
Blood transfusion	225(37.9)	19(67.9)		6(4.7)	3(11.5)	
Admission to ICU	53(8.9)	12(42.9)		29(22.7)	11(42.3)	
Cesarean section	166(27.9)	9(32.1)		39(30.5)	8(30.8)	
Laparotomy other than CS	73(12.3)	4(14.3)		40(31.2)	4(15.4)	

MNM, maternal near miss; MD, Maternal death; MI, mortality index (MD/MD+MNM*100); WHO, World Health Organization; ICU, intensive care unit; CS, cesarean section

As shown in Table 6, coverage of key process indicators ranged from 79% for the use of therapeutic antibiotics in sepsis to 95% for the use of magnesium sulphate in eclampsia. Oxytocin use among women with postpartum hemorrhage was 73%. Mortality index was found to be highest among cases of postpartum hemorrhage (12.5%); and least among sepsis (2.4%) (Table 6).

**Table 6:** Process and outcome indicators related to specific conditions among women with SMO in eastern Ethiopia, 2017.

Indicators	Number	Percentage
**1. Treatment of severe postpartum hemorrhage**		
**Target population: women with severe postpartum hemorrhage**	**77**	
Oxytocin use	46	59.7
Ergometrine	18	23.4
Misoprostol	20	26.0
Other uterotonics	6	7.8
Any of the above uterotonics	56	72.7
Hysterectomy	7	9.1
Proportion of cases with SMO ^a^	24	31.2
Mortality	6	7.8
**2. Anticonvulsants for eclampsia**		
**Target population: women with eclampsia**	**227**	
Magnesium sulfate	215	94.7
Other anticonvulsant	30	13.7
Any anticonvulsant	215	94.7
Proportion of cases with SMO ^a^	26	11.5
Mortality	9	4.0
**3. Prevention of caesarean section related infection**		
Target population: women undergoing caesarean section	325	30.8
Prophylactic antibiotic during caesarean section	316	97.2
**4. Treatment for sepsis**		
**Target population: women with sepsis**	**126**	
Parenteral therapeutic antibiotics	100	79.4
Proportion of cases with SMO ^a^	25	19.8
Mortality	3	2.4
**5. Ruptured uterus**		
**Target population: women with ruptured uterus**	**53**	
Hysterectomy	39	73.6
Proportion of cases with SMO ^a^	39	73.6
Mortality	2	3.8

SMO = severe maternal outcome (MNM + MD).

^**a**^ according to the original WHO MNM tool

## Discussion

We investigated the applicability of the sub-Saharan Africa MNM tool compared to the original WHO tool for use in low-income setting hospitals in eastern Ethiopia. Our study showed that the sub-Saharan Africa criteria identified all the maternal near miss cases identified by the WHO criteria. More importantly, it additionally identified more women with life-threatening complications (including two maternal deaths) which did not fulfil the strict WHO criteria [9,20]. The WHO recommends the use of the more severe cases to avoid burden of data collection and unnecessary inclusions of non-severe cases [8]. However, the mortality index of cases identified by the new classification is 4.5%, indicating severity of the cases.

The major difference in MNM between the two criteria can be attributed to eclampsia, sepsis and difference in the threshold for the number of blood transfusion. In this low-income setting, eclampsia is one of the major underlying cause of maternal death (mortality index of 25.7% among cases fulfilling the WHO criteria). Similarly, of 406 women who received blood products, only nine received five or more units of blood while majority of them received one (229) or two (118) units only (S1 Fig). In the presence of serious shortage of blood, having five or more units of blood for transfusion available is almost impossible in many hospitals in low-income settings [23]. Many of the conditions reported as potentially life-threatening conditions (severe postpartum hemorrhage, severe pre-eclampsia, eclampsia, sepsis, and ruptured uterus) are in fact life-threatening in many low-income settings [19,24–26]. In Ethiopia, death from hemorrhage and eclampsia is so common [27] that their inclusion in MNM will raise awareness to reduce preventable complications and mortality.

Our MNM ratio of 80 per 1000 live births for the adapted criteria was comparable with a previous study from Ethiopia which used the disease-based criteria (78.9) [18]. However, it is higher than findings from other studies using more comparable adapted MNM tools in Tanzania (23.6), Malawi (10.2), and Rwanda (21.5) [11,28,29]. Compared to these studies, our study was conducted in urban centers including a tertiary referral hospital where the majority of women with complications are treated. Comparing our finding of 17 per 1000 live births according to the WHO criteria with other studies using the WHO tool showed that our finding is higher than findings from Addis Ababa, Ethiopia (8), Zanzibar (6.7), Nigeria (15.8) and South Africa (4.4) [12,13,17,30]. This may be related to low institutional delivery rates in our setting, where more births occur outside hospitals [31]. On the other hand, it is lower than the findings from Ghana (28.6) and Rwanda (110) [15,32]. Differences in the study population or quality of care may play a role.

The strength of our study is the use of prospective identification of cases and data collection over a long period of time. However, our findings are limited by the fact that this is the first study to apply the adapted criteria in real clinical settings. We did sub-group analysis of MNM outcomes for the adapted and the original WHO tool to minimize the limitation and compare with other studies as appropriate. Although most MNM and deaths are better identified in facilities [33,34], the denominator (live births) could be low because of high home births in Ethiopia [31]. Our study was conducted in a tertiary and regional hospital in one district and, therefore, findings may not be generalizable. We are unable to comment on timeliness of treatments and delays associated with management since the time between decision and actual treatment is rarely documented. Because of poor documentation, majority of sociodemographic characteristics (income, educational status, partner’s status, and occupation) affecting treatment seeking or outcome were not collected. Our follow up is also limited up to discharge of the women, and therefore cases occurring after discharge until 42 days may be missed—especially if not re-admitted in both hospitals.

In conclusion, the sub-Saharan Africa criteria functioned well in identifying all maternal deaths and all MNM cases identified by the WHO criteria. The tool additionally captured MNM cases—that are common causes of maternal morbidity and mortality—that were missed when applying the WHO MNM tool. Although common criteria for MNM may enable comparisons across settings, the local context must be taken into account without creating too many different adaptations of standardized criteria [9,28]. The WHO criteria failed to identify two third of women with severe acute maternal morbidity and more than one third of maternal deaths even in high-income settings, the Netherlands [35]. The need for refined MNM criteria with limited set of interventions- and organ dysfunction-based criteria was previously reported [36]. Therefore, use of the sub-Saharan Africa MNM tool should be encouraged for use in low-income settings with limited personnel and sophisticated laboratory. Similar studies are required to assess broader performance of the tool and its applicability in other low-income settings.

## Supporting information

S1 Fig Differences in MNM inclusion based on threshold for blood transfusion. (TIFF)
