# Supplementary figures and images for: Severe maternal outcomes in eastern Ethiopia: Application of the adapted maternal near miss tool
Source: PLoS One. 2018 Nov 14;13(11):e0207350. doi: 10.1371/journal.pone.0207350 (PMC6235311; doi:10.1371/journal.pone.0207350)

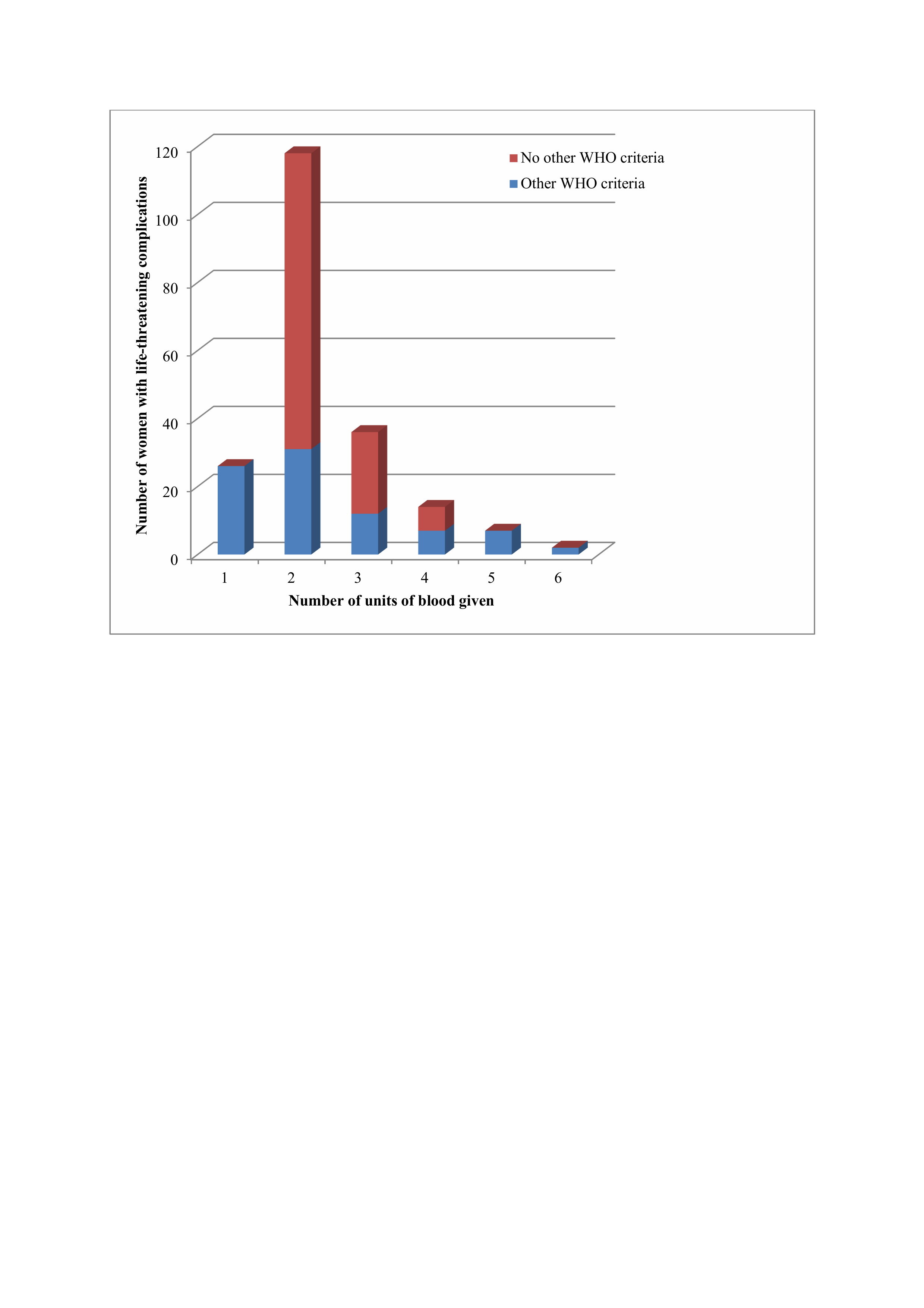

Supplement: S1 Fig — (TIFF) [file pone.0207350.s001.tiff]
